# Conceptual Framework of Mentoring in Low- and Middle-Income Countries to Advance Global Health

**DOI:** 10.4269/ajtmh.18-0557

**Published:** 2018-11-14

**Authors:** Shailendra Prasad, Elizabeth Sopdie, David Meya, Anna Kalbarczyk, Patricia J. Garcia

**Affiliations:** 1 Center for Global Health and Social Responsibility, University of Minnesota, Minneapolis, Minnesota;; 2 College of Education and Human Development, University of Minnesota, Minneapolis, Minnesota;; 3 Infectious Disease Institute, Makerere University, Kampala, Uganda;; 4 Center for Global Health, Johns Hopkins University, Baltimore, Maryland;; 5 School of Public Health and Administration, Cayetano Heredia University, Lima, Peru

## Abstract

Although mentoring is not a common practice in low- and middle-income countries (LMICs), there is a strong need for it. Conceptual frameworks provide the structure to design, study, and problem-solve complex phenomena. Following four workshops in South America, Asia, and Africa, and borrowing on theoretical models from higher education, this article proposes two conceptual frameworks of mentoring in LMICs. In the first model, we propose to focus the mentor–mentee relationship and interactions, and in the second, we look at mentoring activities from a mentees’ perspective. Our models emphasize the importance of an ongoing dynamic between the mentor and mentee that is mutually beneficial. It also emphasizes the need for institutions to create enabling environments that encourage mentorship. We expect that these frameworks will help LMIC institutions to design new mentoring programs, clarify expectations, and analyze problems with existing mentoring programs. Our models, while being framed in the context of global health, have the potential for wider application geographically and across disciplines.

## Introduction

Various adult learning theories have been proposed to understand the complex processes of higher education.^1^ Whereas these theories look at different aspects of knowledge and skills acquisition, the ultimate task of a learner is to achieve mastery in the chosen field while being a lifelong learner.^2^ The progression to mastering skills for lifelong learning occurs with a transition from rule-based behavior to a context-based one that enables adaptive learning for emergent knowledge.^3^ This progression of mastery occurs in the context of increasing changes within individual fields. The field of global health adds further layers of complexity to learning with changing paradigms of diseases, systems, and inter- and trans-disciplinarity, including health and non-health sciences, and cross-cultural challenges.^4^

This article proposes a conceptual model of mentoring, particularly for the low- and middle-income country (LMIC) setting. It takes into consideration the unique challenges of working across cultures and disciplines, and looks at resource-limited settings in which most mentoring programs are nascent. We draw on the literature from higher education and from cross-cultural studies to provide a framework for designing and evaluating mentorship programs. Although mentoring occurs in informal ways without conceptual models in various settings, including in LMICs, we propose these conceptual models as a framework to let groups of mentors organize their work, generate new ideas, and develop programs within their institutions. The conceptual model also sets expectations for mentees to use these programs to advance their careers and global health. This conceptual model originated from the four aforementioned Fogarty International Center Global Health Program for Fellows and Scholar consortia members “Mentoring the Mentors in Global Health Research” workshops at LMIC institutions detailed earlier in this special issue.^5^

### Critical role of a mentor.

The ongoing development of knowledge in each trainee from early training through postdoctoral and early faculty positions is shaped by core adult learning principles: the learner’s need to know, self-concept, experiences, readiness to learn, orientation to learning, and motivation.^6^ Incorporation of these principles can lead to “transformative” learning which finds meaning from experience, thus providing a basis for action.^7^ Learners need to critically reflect and engage in a deep conversation or discourse with their life and work experiences, beyond engagement in formal curricular elements, to support transformational learning, but are often ill prepared to undertake this type of reflection without guidance from a more experienced professional.^8^ This guidance often falls to a mentor, whose essential function is to prompt critical reflection in student learning.^9^ Mentorship has been defined as—“*an experienced highly regarded empathic person (the mentor) guides another individual (the mentee) in the development and re-examination of his or her own ideas, learning, personal, and professional development.*”^10^

### Why conceptual models?

Conceptual frameworks represent ways of thinking about a problem or ways of representing the inner workings of complex phenomena.^11^ These frameworks, besides providing a reference point and structure for discussion of current literature, methodology, and results, identify the boundaries of the work and enable individuals and teams to move beyond descriptions of the “what” to explanations of “why” and “how.”^12^ Conceptual models have been used in many fields and help to define aspects of a complex issue. For example, if a surgical training program is noticing poor surgical skills, using the theory of expertise, it would be possible to design training that considers appropriate resource utilization, adequate effort, and motivation of the participants.^13^ Similarly, conceptual frameworks based on self-determination theory, proposed by Deci and Ryan, have helped shape programs that evaluate motivation among learners.^14^

### Tenets of mentorship.

Conversations about mentoring are relatively new in health sciences literature and it is important to consider elements from student development and career guidance literature when proposing a framework to guide trainee learning. Conceptual models have been proposed in student development since the 1960s and continue to develop as higher education disaggregates student populations to gather more nuanced data about individual growth. Early theories followed psychological theories of development, which suggested that students develop in linear or sequential stages. These theories attempted to answer questions related to what end student development is directed and what skills are necessary to address complex problems in society. These include intellectual capacities, values, types of learning, ways to foster lifelong learning, and participating in the global community.^15^

The field of student development has evolved from linear models to focus more on how environmental factors might influence development.^16,17^ Most theories share assumptions about the social construction of identity and the importance of considering environmental influences or context as a complex system that affects behavior, attitudes, and cognition.^17^ This focus on learning environment places responsibilities on the institutions of learning, although the individual student remains at the center of many of these models.^15^

Many of these student development frameworks focus on undergraduate students, not graduate or professional students.^18^ In graduate and professional schools, the additional component of socialization is an important factor that needs to be taken into consideration.^19^ Socialization occurs when the student integrates with the culture, values, and norms of a profession and is “the processes through which individuals gain the knowledge, skills, and values necessary for successful entry into a professional career requiring the advanced level of specialized knowledge and skills.”^20^ In the health sciences, where apprenticeship is the primary model of education, there has been particular emphasis on the importance of this form of socialization.^21^ While considering mentoring in LMIC settings, it is important to retain socialization as an integral part of the conceptual model.

### Mentorship as leadership.

Mentoring has also been described as an essential part of graduate education, and has been emphasized particularly to teach ethical responsibilities,^22^ while progressing toward degree completion, developing research and training skills, and ensuring employment opportunities.^23,24^ Mentoring ideally combines development of technical skills along with individual development.^16^ This in essence would be a model of “situational leadership,” where there is no “best” leadership, but rather leadership is considered to be task- and situation-relevant.^25^ This would entail the mentor to switch between the four leadership behavior styles of directing, coaching, supporting, and delegating.^25^ The mentor would switch between these styles while sustaining a common vision with the mentee and the institution.^26^

Although attributes that mentors should possess have been described, there is lack of clarity in conceptual models regarding mentoring.^27^ Criticism of the various student development models is that very few of them describe learner-centered approaches^28^ and that many of the theories revolve around a white male standard or “norm.”^20^ In addition, many of these models, while informative, are not directly applicable to the context of global health learning in which learners interact with emerging and possible cross-cultural situations across multiple disciplines.

### Unique factors to consider in LMICs.

Research in LMICs goes far beyond the institutional boundary to include additional social, political, economic, national, regional, and global influences. Current models do not capture the complexity and variation of context as well as the critical nature of the relationship between the mentor and mentee in the LMIC context. In this sense, global health research and education can be considered “boundary work” that takes place in between and across disciplines and requires collaboration. Mobility, technology, and globalization have blurred lines and boundaries between organizational units and illuminated interdependent networks between sectors,^29^ leading to structural changes.^26,30^ These collaborations generate new practices, rules, and technologies that can diffuse beyond the boundaries of the collaborative group and be adopted by other organizations or even become new institutions themselves.^31^ The conversation about mentoring in LMICs should acknowledge this boundary work and the cultural and contextual factors involved.

## Conceptual Framework for Mentoring

**Figure 1.**
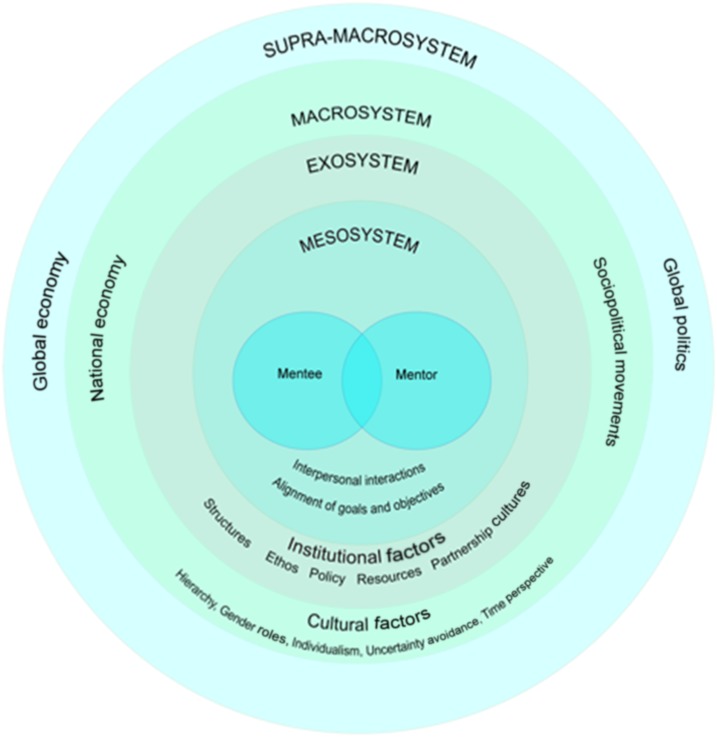
Systems of interaction between the mentor and mentee. This figure appears in color at www.ajtmh.org.

**Figure 2.**
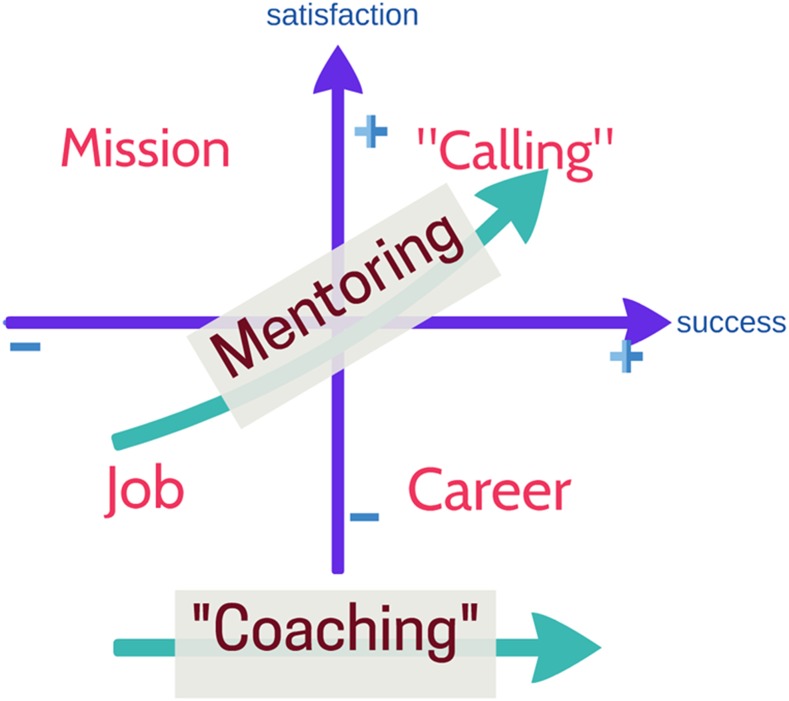
Success and satisfaction in mentor–mentee relationships. This figure appears in color at www.ajtmh.org.

To address criticisms of the lack of mentee-centered approaches, go beyond the developed country framework, and capture the complexity of working in LMICs as mentioned in the prior section, we propose two conceptual models for mentoring. In the first model, we propose to focus the mentor–mentee relationship and interactions (Figure 1), and in the second, we look at mentoring activities from a mentees’ perspective (Figure 2).

### Context in which mentoring occurs.

Mentorship, with its need to sustain a common vision and its emphasis on growth,^31^ inherently is a manifestation of leadership. Through the use of “situational leadership” as a model, we can shift the perspective from mentorship as performance to mentorship as a dynamic interaction with ongoing learning for both the mentor and mentee.^32^ For the mentor–mentee relationship, we borrow from the Psycho-Ecological Systems Model^33^ which primarily integrates Bronfenbrenner’s Ecological Systems Model.^34^ In contrast to both the Psycho-Ecological Systems Model and the Ecological Systems Model, at the center of our conceptual framework is the interaction and relationship between the mentor and the mentee. The contextual factors of these interactions that are particularly important to consider in global health are represented in the concentric circles. The “mesosystem” in the model includes the immediate factors in the microenvironments of the mentor and mentee that influence their interactions, the “exosystem” are the larger institutional factors where mentorship take place, the “macrosystem” represents the societal factors, and the “supra-macrosystem,” as described in the psycho-ecological systems model, represents global and international influences.

At the center of this model, the interactions between the mentee and mentor are influenced by individual factors and behavior of both the mentor and mentee, such as gender, age, religious congruencies, cultural backgrounds, ethnicities, vulnerabilities, resources, and worldview. One of the important factors that predicts success in this is the “click”—the connection between the mentor and mentee.^35^ The mesosystem is in effect a “system of the microsystems.” In our model, we posit this to be the interactions that the mentor and mentee have with their surroundings, family, different social agencies, etc.^33^ The exosystem reflects the institutional factors that include the current structures, available institutional resources, organizational ethos, and policy and partnership cultures. It is important to consider these both from the mentor’s and the mentee’s institutional perspective, as the social interactions (microsystems) that they have within the institution would influence the mentorship interactions.

At the macrosystem level, we include the cultural/societal factors. These include sociopolitical movements, national economy, and cultural factors. Our model could be used to understand the context of mentor–mentee interactions when both mentor and mentee are from similar cultural backgrounds and when they are from vastly different cultural backgrounds. We use Hoftede’s cultural dimensions as important tenets at this level.^36^ Hofstede describes five dimensions of culture: 1) *Power distance (Hierarchy)*, 2) *individualism versus collective*, 3) *uncertainty avoidance*, 4) *gender roles*, and 5) *time perspective* (long-term versus short-term orientation).^37^ Hierarchical issues are crucial in cross-cultural mentor–mentee relationship because expectations based on the native culture of the individual participants may create different expectations. This is particularly important to consider when the mentor’s role is beyond that of a task-oriented coach and focuses on nurturing transformative learning and critical reflection (Figure 2). *Individualism versus collective* perspectives would inform the outlook toward global health work and the resilience and support systems that one can use when needed. *Uncertainty avoidance* is “society’s tolerance toward ambiguity.”^36^ In global health activities, where the science of the work can be demanding, and the circumstances in which the work done can be challenging, this tolerance toward ambiguity can be an asset, provided that it does not lead to complacency. *Gender roles* come into play when there is gender discordance between the mentor and mentee or if the work involves gender discordant aspects. The *time perspective* associates connections of the past with the current and future actions.^36^ Each of these dimensions has to be considered at the mentor and the mentee level, and when mentors or mentees do not share the cultural perspectives of the other, it may contribute to unrealistic or crossed expectations in the mentor–mentee relationship.

The supra-macrosystem includes the global economy and sociopolitical factors that may affect the global health work. It forms the context in which priorities in global health are expressed (e.g., Sustainable Development Goals) and can influence the work being done as well as the learning that occurs for the mentee. Although its effect on the individual mentor–mentee relationship is minimal, it is important to consider that elements of the supra-macrosystem could play a major role in influencing funded projects, deadlines, and priorities.

### Mentee-centered mentoring.

Placing the mentee at the center of the mentoring relationship is a key component of our conceptual model. As such, the interactions between the mentor and the mentee are at the center of the model. Figure 2 explores the dimensions at the center of Figure 1. It represents where the mentee is with respect to “success,” that is, mentee-defined and tangible, and “satisfaction,” an internal feeling that the mentee experiences. The four quadrants in the figure represent different stations that a mentee can exist and is dynamic based on the degree of success and satisfaction that the mentee experiences over time. When there is low success and satisfaction, the mentee is doing a “job.” This could be manifested as the mentee being a disinterested participant in the work. When there is a high degree of success but low satisfaction, we consider it a “career” in this paradigm. Whereas “mission” represents a high degree of satisfaction, the low “success” may lead to a gradual drift to the “job” station. When the mentee experiences both a high degree of success and satisfaction, we label it as a “calling,” which we believe represents the ideal station for a mentee to attain. These terms are not used pejoratively and these are not unique to LMICs, nor to any particular discipline.

### Coaching versus mentoring.

Historically, in the health sciences, coaching and mentoring have been used synonymously. However, we differentiate these two concepts: coaching is a task-oriented, skill acquisition that is generally short term and performance driven. As indicated in Figure 2, coaching is essentially moving the mentee along the “success” axis. By contrast, mentoring is person oriented, rooted in relationships, and is development driven. This is represented as a way to nudge a mentee into the “calling” quadrant that is indicated by high degrees of both success and satisfaction. Achieving success would be further incentive for continued lifelong learning along with finding the “joy of discovery” that transformative learning would entail. Two other terms that came up in our workshops, in the context of mentorship, were “supervision” and “sponsorship.” Supervision refers to ensuring appropriate completion of tasks of the mentee and is seen as an extension of the “coaching” function. When the mentor promotes or advocates for the mentee, with the intention of ensuring good positions for the mentee, that is sponsorship.

### The practice of mentorship.

The practice of structured mentorship with sufficient institutional support is relatively new in higher education even in high-income countries, and current literature discusses attributes that are important for mentors to have to ensure effective mentorship.^38–41^ Our conceptual model creates a framework for discussion and analysis of mentorship across various LMIC settings including academic and research institutions. Using a psychosocial ecological framework, we have placed the interactions between the mentor and mentee at the heart of the conceptual framework surrounded by institutional, sociocultural, political, and global influences. Our framework acknowledges that, although presented as a nested model, the influences can be crosscutting between the various system levels. These considerations are particularly important in the global health context where there is a high likelihood of cross-cultural interactions. We feel that this conceptual framework can help inform the creation of structures and processes within institutions that help guide the development of effective mentorship programs.

Literature on mentorship places the onus on the mentee to initiate, maintain, and use mentorship interactions to benefit one’s career.^42,43^ We highlight mentorship as a dynamic between the mentor and mentee, and this ongoing interaction indicates a reciprocity that should be beneficial to both members of this dyad. The emphasis on being mentee-centric is crucial as that shifts from a passive transfer of information from the mentor to the mentee to one of a development curriculum for the mentee.^44^ As such, the onus now shifts to both the mentor and mentees to maintain the interactions and to the institution to create an enabling environment to develop a foundation that encourages mentorship through incentives for promotion (mentor), creation of training programs for mentorship (both), ensure successful growth of the mentee, and overall development of the institution.

Historically, most of the literature on mentorship has emphasized achieving tasks, which we argue in this article is better defined as “coaching.” This is not to minimize the need and importance of coaching. Coaching is possibly inherent in mentoring situations, particularly in the global health arena. Our contention is that ensuring that the mentee considers the satisfaction axis as he/she is achieving academic success is key to the purpose of a mentor. Isolation and unsatisfying relationships with colleagues are strongly associated with burnout.^45^ A well nurtured mentoring relationship that emphasizes and nudges the mentee up the satisfaction axis may decrease burnout both in the mentee and the mentor; further research is required to test this hypothesis. Mentorship in research brings some added aspects of socialization of the mentee in the field, the joint discovery through the research being conducted, and modeling of research ethics by the mentor. Low- and middle-income countries’ institutions, as in many high-income country institutions, are going through changes. This is of particular importance because the generational differences between the mentor and mentee can add to the complexity of the interactions. The key issue with the conceptual model is to assure that there is a progression of the mentee in the academic field of choice. By ensuring the mentees achieve satisfaction in the work being done, while achieving academic success, our model suggests that LMIC institutions can protect their most precious resource—the personnel.

### How to use the conceptual model.

We propose the use of our conceptual models in the following ways: 1) design new mentoring programs and institutionalize them; 2) analyze structural challenges in setting up mentorship programs; 3) analyze challenges in mentor–mentee interactions; 4) create a template to train mentors in comprehensive mentorship activities; and 5) set expectations for mentees. Educational institutions, as in other entrenched organizations, are often hesitant to take risk and create new structures.^46^ The internal forces within institutions are inertial, and external forces, including global health research, can be disruptive. In such a dynamic, while designing new programs, careful consideration of the system factors that exist outside the mentor–mentee relationship will allow for creating deliberate structures to overcome structural barriers. For example, creation of peer-mentor groups to decrease hierarchical structures.^47^ The cultural domains depicted in the macrosystem level should allow for effective pairing and problem-solving when concerns arise that interfere with effective communication between mentors and mentees. Recognizing the need to emphasize the developmental journey of the mentee would lead to more effective mentor training and exposure to the conceptual models would allow mentees to effectively activate, nurture, and use mentor–mentee interactions to enable transformative learning as lifelong students.

## Conclusion

Mentoring is a much needed part of higher education. Our conceptual model provides a structure to help plan and analyze mentorship programs. Whereas we frame this in the context of global health, particularly in the LMIC context, the conceptual model has potential wider application geographically and across disciplines. We contend that institutional support to create mentorship models is imperative. The next article in this special issue will elaborate on the mentoring framework by proposing core competencies essential for mentoring in LMICs.
